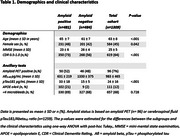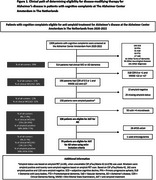# ‘Real‐world’ eligibility for anti‐amyloid treatment in a tertiary memory clinic setting

**DOI:** 10.1002/alz70860_102927

**Published:** 2025-12-23

**Authors:** Sinthujah Vigneswaran, Everard G.B. Vijverberg, Frederik Barkhof, Elsmarieke van de Giessen, Afina W. Lemstra, Yolande A.L. Pijnenburg, Charlotte E. Teunissen, Wiesje M. van der Flier, Argonde C. van Harten

**Affiliations:** ^1^ Alzheimer Center, Department of Neurology, Amsterdam UMC, Vrije Universiteit Amsterdam, Amsterdam Neuroscience, Amsterdam, Netherlands; ^2^ Neurochemistry Laboratory, Department of Laboratory Medicine, Amsterdam Neuroscience, Amsterdam UMC, Vrije Universiteit Amsterdam, Amsterdam UMC, Amsterdam, the Netherlands., Amsterdam, Netherlands; ^3^ Amsterdam Neuroscience, Neurodegeneration, Amsterdam, Netherlands; ^4^ Department of Radiology and Nuclear Medicine, Vrije Universiteit Amsterdam, Amsterdam UMC location VUmc, Amsterdam, Netherlands; ^5^ Department of Radiology and Nuclear Medicine, Vrije Universiteit Amsterdam, Amsterdam University Medical Center, location VUmc, Amsterdam, Netherlands

## Abstract

**Background:**

Recent approvals of anti‐amyloid therapies (AAT) for Alzheimer's disease (AD) in various countries highlight the need to assess patient eligibility due to potentially high costs. Estimates suggest 1–18% of patients may qualify based on biomarkers, but real‐world data remain limited. This study evaluated eligibility for AAT in a tertiary memory clinic from 2020 to 2022 using lecanemab criteria.

**Method:**

We included 1309 patients (63 ± 8 years, 45% women, Mini‐Mental State Examination (MMSE) 25 ± 5) from Alzheimer Center Amsterdam (2020–2022) who underwent standard diagnostic workup (Table 1). Eligibility for AAT followed U.S. Food and Drug Administration provided prescribing information and the appropriate use criteria for lecanamab, along with the APOE criteria as defined by the European Medicines Agency. Patients were eligible (1) when clinical diagnosis of mild cognitive impairment (MCI) or AD with (2) a Clinical Dementia Rating (CDR) of 0.5 or 1.0 and a MMSE ≥22 and ≤27, when they (3) were amyloid positive based on cerebrospinal fluid or positron emission tomography and (4) <4 microbleeds on brain magnetic resonance imaging, excluding APOE ε4/ε4 carriers and anticoagulant users. For patients with MCI/AD and unknown amyloid status, we calculated a range, assuming all could be either amyloid positive (upper limit) or amyloid negative (lower limit).

**Result:**

Among 1309 memory clinic patients, 514 (39% of all‐comers) had a clinical diagnosis of MCI or AD. Of these, 196 (15% of all‐comers, 38% of MCI/AD) met inclusion criteria for CDR and MMSE (Figure 1). Of these 196, 158 (12% of all‐comers, 31% of MCI/AD) were amyloid positive and 25 had an unknown status. Among amyloid‐positive patients, 50 had more than four microbleeds, leaving 108 (8% (range 7‐9%) of all‐comers, 21% (18‐24%) of MCI/AD) eligible for AAT. After excluding APOE ε4 homozygotes and anticoagulant users, 79 (6% (5‐7%) of all‐comers, 15% (14‐17%) of MCI/AD) remained eligible.

**Conclusion:**

In our tertiary memory clinic, 8% of all‐comers and 21% of those clinically diagnosed with MCI or AD met in‐ and exclusion criteria for AAT. This information can be taken into account when estimating the preparedness of the health care system and budget‐impact analyses for these drugs.